# RNA Splicing of *FLC* Modulates the Transition to Flowering

**DOI:** 10.3389/fpls.2019.01625

**Published:** 2019-12-17

**Authors:** Hao-Dong Qi, Yi Lin, Qiu-Ping Ren, Yu-Yi Wang, Feng Xiong, Xiu-Ling Wang

**Affiliations:** ^1^National Key Laboratory of Crop Biology, College of Life Sciences, Shandong Agricultural University, Taian, China; ^2^College of Agronomy, Liaocheng University, Liaocheng, China

**Keywords:** alternative splicing, FLOWERING LOCUS C, COOLAIR, flowering transition, splicing factor, vernalization

## Abstract

Flowering is a critical stage of plant development and is closely correlated with seed production and crop yield. Flowering transition is regulated by complex genetic networks in response to endogenous and environmental signals. FLOWERING LOCUS C (FLC) is a central repressor in the flowering transition of *Arabidopsis thaliana*. The regulation of *FLC* expression is well studied at transcriptional and post-transcriptional levels. A subset of antisense transcripts from *FLC* locus, collectively termed cold-induced long antisense intragenic RNAs (*COOLAIR*), repress *FLC* expression under cold exposure. Recent studies have provided important insights into the alternative splicing of *COOLAIR* and *FLC* sense transcripts in response to developmental and environmental cues. Herein, at the 20th anniversary of *FLC* functional identification, we summarise new research advances in the alternative splicing of *FLC* sense and antisense transcripts that regulates flowering.

## Highlights

FLOWERING LOCUS C (FLC) is a key repressor in flowering transition. The alternative splicing of *FLC* sense and antisense transcripts regulated by external and internal cues modulates flowering transition.

## Introduction

RNA splicing is a critical step in the post-transcriptional regulation of gene expression. This process occurs by removing intronic sequences and joining exons by spliceosome and numerous splicing factors (Jurica and Moore, 2003; Wahl et al., 2009; Matera and Wang, 2014; Vosseberg and Snel, 2017). Spliceosome is a highly dynamic ribonucleoprotein complex that catalyses RNA splicing and is composed of five small nuclear ribonucleoprotein (snRNP) particles (U1, U2, U4/U6 and U5) (Will and Lührmann, 2011; Fica et al., 2019). Splicing factors are one of the key determinants as accessory non-snRNP proteins regulating RNA splicing (Cho et al., 2011; Liu et al., 2016; Long et al., 2019; Wang et al., 2019; Xiong et al., 2019a; Xiong et al., 2019b). A pre-mRNA may undergo different splicing patterns, creating various mature transcripts that encode distinct functional proteins (Samach et al., 2011; Wang et al., 2015; Zhu et al., 2017; Lockhart, 2018; Okumoto et al., 2018). This phenomenon is called alternative splicing (AS).

AS fulfils important biological functions in plants, such as flowering transition and flower development (Wang et al., 2014; Melzer, 2017; Rodríguez-Cazorla et al., 2018; Park et al., 2019a; Park et al., 2019b; Wang et al., 2019). AS is also implicated in plant response to circadian rhythm regulation (Filichkin and Mockler, 2012), phytohormone (Hrtyan et al., 2015; Wang et al., 2015; Zhu et al., 2017; Xiong et al., 2019a), ambient temperature (Verhage et al., 2017) and abiotic and biotic stresses (Lyons and Kazan, 2016; Huang et al., 2017; Mei et al., 2017; Shang et al., 2017; Laloum et al., 2018). These cues are all important for flowering transition; therefore, AS plays multiple roles in flowering by integrating endogenous developmental and exogenous environmental signals. The flowering inhibitor gene flowering locus c* (FLC)* encodes a MADS-box transcription factor and is a key regulator of vernalisation and autonomous pathways in *Arabidopsis* and related species (Michaels and Amasino, 1999; Sheldon et al., 1999; Sheldon et al., 2000; Chen et al., 2019; Coupland, 2019). FLC inhibits flowering by repressing the expression of a subset of key genes in promoting flowering, such as flowering locus t (*FT*), suppressor of overexpression of constans 1 (*SOC1*) and target of flc and svp1* (TFS1)* (Helliwell et al., 2006; Searle et al., 2006; Luo et al., 2019; Richter et al., 2019). Thus, *FLC* regulation is central for flowering at the transcriptional, post-transcriptional and post-translational levels (Michaels et al., 2003; Lempe et al., 2005; Li et al., 2016; Kwak et al., 2017; Whittaker and Dean, 2017; Xiong et al., 2019a). The AS of *FLC* sense and antisense transcripts is required for flowering transition in *Arabidopsis* and other dicots (Michaels and Amasino, 1999; Sheldon et al., 1999; Helliwell et al., 2006; Yuan et al., 2009; Wu et al., 2012).

Twenty years ago, the works of Michaels and Amasino (1999), and Sheldon et al. (1999) provided a first glimpse of the central functions of *FLC* in flowering and the molecular basis of *FLC* in vernalisation (Coupland, 2019). Subsequent research works have demonstrated the regulation of *FLC* at the transcriptional and post-transcriptional levels, especially the epigenetic silencing of *FLC* by histone methylation in vernalisation, and the splicing regulation of *FLC* sense and antisense transcripts by splice factors (Bastow et al., 2004; Liu et al., 2007; Liu et al., 2010; Marquardt et al., 2014). In this review, we describe the current understanding of the AS of *FLC* sense and antisense transcripts in modulating flowering time and the splice factors involved in these processes.

## As of *FLC* Sense Transcripts Mediates Flowering Time

**Figure 1:**
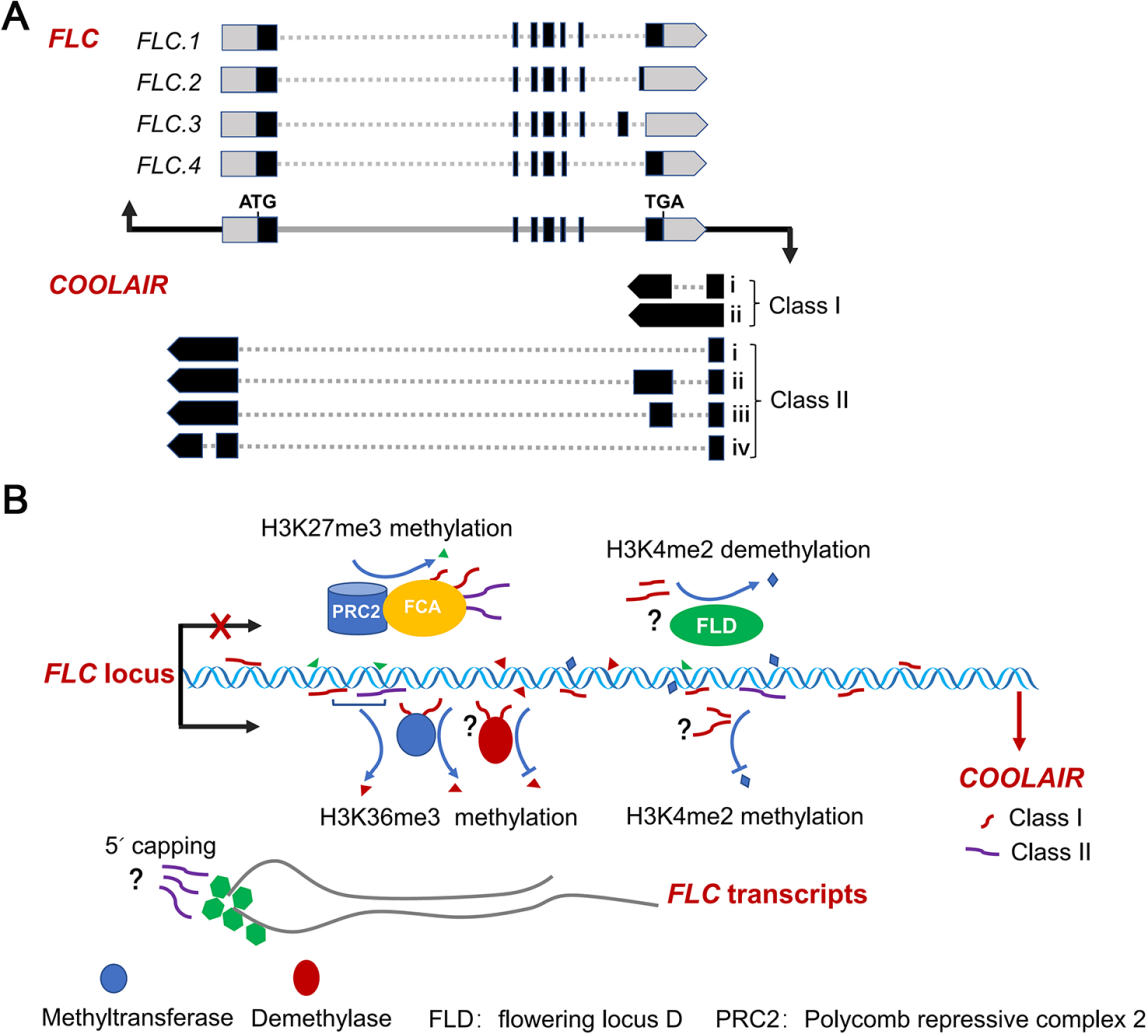
Splicing patterns of *FLC* sense and antisense transcripts, and AS of *FLC* antisense transcripts regulating *FLC* expression. **(A)** Schematic diagram of the splicing patterns of *FLC* sense and antisense transcripts at *FLC* locus in *Arabidopsis*. Black rectangles, gray dash lines and boxes indicate exons, introns and non-translated regions of *FLC* RNAs, respectively. Gray and black lines represent the genes and promoter regions of *FLC* and *COOLAIR* loci, respectively. **(B)** AS variants of *COOLAIR* transcripts effect *FLC* expression. Nascent *COOLAIR* RNAs are physically associated with the *FLC* locus to regulate the switching of chromatin states (Csorba et al., 2014; Li et al., 2015; Rosa et al., 2016). Two classes of *COOLAIR* transcripts bind to FCA and recruit PRC2 complex to *FLC* locus (Tian et al., 2019). The class I variants with proximal polyadenylation and the proximal–distal ratio of *COOLAIR* transcripts effect the histone methylation of H3K4me2, H3K36me3 and H3K27me3 (Marquardt et al., 2014; Berry and Dean, 2015; Xiao et al., 2015; Rosa et al., 2016). The class II *COOLAIR* transcripts with distal polyadenylation influence the degree of capping of the *FLC* nascent transcripts (Li et al., 2015).

*Arabidopsis* accessions exhibit markedly different flowering behaviour from different environments. The AS of *FLC* sense transcripts in these accessions generate multiple splice isoforms (**Figure 1A**). The naturally occurring splice variants of *FLC* are related to different vernalisation responses of various *Arabidopsis* accessions (Bloomer and Dean, 2017). *Arabidopsis* ecotype Bur-0 is late flowering and vernalisation insensitive. *FLC* cDNA from Bur-0 contains 64 bp of intron sequence immediately upstream of exon 7, causing a mutation at the final position of intron 6. The 64 bp intron retention causes a frame shift and a premature stop codon in *FLC* cDNA. Thus, the encoded FLC lacking the C-terminal 33 amino acid residues is a null function protein in Bur-0 (Werner et al., 2005). Similarly, in variations of Cen-0 and Cal-0, alternative splice acceptor sites in the last exon and last intron are used, respectively. These aberrant splicing forms all lead to a frame shift of cDNA sequences and severely compromise protein function (Lempe et al., 2005). In Col-0 *Arabidopsis*, there are several additional splicing variants from *FLC* locus besides the canonical transcript (Severing et al., 2012; Park et al., 2019a); however, their roles in flowering transition remains to be investigated. An additional shorter transcript from *FLC* locus is induced after vernalisation treatment for 15 days in Est-0 and Le-0 ecotypes, which is not observed at normal temperature (Caicedo et al., 2004). Whether this short transcript is involved in vernalisation-induced flowering in *Arabidopsis* is unknown.

In other plant species, the naturally occurring splice variants of the *FLC* locus also reveal that *FLC* AS is important for the control of flowering time. For example, four different transcripts of the *FLC* homologs in *Brassica rapa BrFLC1* and *BrFLC2* have been identified from naturally occurring splicing mutations (Yuan et al., 2009; Zhao et al., 2010; Wu et al., 2012). These different splice types of *BrFLCs* are significantly associated with a natural variation of flowering time in different germplasms of *B. rapa*. Additionally, differential splicing variants of *BnFLC.A3b*, a *FLC* homolog in *B. napus*, have been observed in leaves at the seedling stage between winter cultivar Tapidor and semi-winter cultivar Ningyou7. The transcripts from Tapidor are usually spliced canonically, but numerous incompletely spliced transcripts have been identified in Ningyou7, causing decreased functional transcripts (Zou et al., 2012). In tetraploid *Capsella bursa-pastoris*, splice site polymorphisms in the *FLC* loci create different transcripts which are nonfunctional (Slotte et al., 2009). These findings partially explain the differential flowering times of *C. bursa-pastoris* from different districts. A natural splicing site mutation in the *BrpFLC1* gene causes early flowering in the cultivated variety of purple flowering stalk (*Bras. campestris L. ssp. chinensis* var. *purpurea*) compared with that of pakchoi (*Bras. campestris ssp. chinensis* var. *communis*) (Hu et al., 2011). Additionally, in an early flowering trifoliate orange (*Poncirus trifoliata*) mutant, five alternatively spliced transcripts of *PtFLC* have been identified; furthermore, their abundances are variable at the juvenile and adult stages, suggesting that the AS of *PtFLC* is related to flowering time (Zhang et al., 2009). Therefore, the AS of *FLC* is a target of natural selection for flowering regulation under natural conditions.

## As of *FLC* Antisense Transcripts Regulates *FLC* Expression

*COOLAIR* is a set of long noncoding RNAs expressed at the *FLC* locus in the antisense direction that was first identified in *Arabidopsis* (Liu et al., 2007; Swiezewski et al., 2007; Swiezewski et al., 2009; Ietswaart et al., 2012). *COOLAIR* RNAs functionally repress *FLC* sense expression at an early stage of cold exposure (vernalisation) *via* different ways, such as by directly associating with *FLC* chromatin or affecting H3K36me3 and H3K27me3 levels (Csorba et al., 2014; Berry and Dean, 2015; Rosa et al., 2016; Pajoro et al., 2017; Whittaker and Dean, 2017; Tian et al., 2019) (**Figure 1B**). The AS of antisense transcripts generated from the *FLC* locus produces two main classes of *COOLAIR* isoforms, terminating at proximal (sense intron 6, class I) and distal (sense promoter, class II) sites of *FLC* locus (Liu et al., 2007; Swiezewski et al., 2009; Marquardt et al., 2014). Each class contains several subclasses produced by AS *associated with the usage of alternative 3′polyadenylation* in spliced variants (**Figure 1A**). These multiple spliced variants with alternative polyadenylation are linked to different *FLC* expression states. Class I *COOLAIR* RNAs with proximal polyadenylation are associated with *FLC* repression (Liu et al., 2007; Swiezewski et al., 2009; Liu et al., 2010; Hornyik et al., 2010; Csorba et al., 2014). By contrast, class II *COOLAIR* transcripts are associated with high *FLC* expression levels. These *COOLAIR* RNAs with distal polyadenylation affect the capping of *FLC* nascent transcripts (Li et al., 2015).

The efficient splicing of class I intron promotes proximal polyadenylation in the antisense transcripts of *FLC*. As a result, this proximal polyadenylation inhibits the transcription of *FLC* through triggering H3K4me2 demethylation in the *FLC* locus (Marquardt et al., 2014). A few 3′-end processing factors containing RNA-recognition motifs, such as flowering control locus a (FCA) and FPA, glycine-rich rna-binding protein7 (GRP7), cleavage stimulation factor 64 (CstF64), CstF77, and nuclear speckle RNA binding protein a (NSRa), affect the splice site selection and polyadenylation site usage of *COOLAIR* transcripts, leading to an altered ratio of proximal–distal spliced variants (Liu et al., 2007; Streitner et al., 2008; Liu et al., 2010; Hornyik et al., 2010; Streitner et al., 2012; Xiao et al., 2015; Bazin et al., 2018). Loss-of-function mutations in these factors decrease or increase the usage of *COOLAIR* proximal polyadenylation site, leading to upregulated or downregulated *FLC* transcription.

The AS of *COOLAIR* is altered by environmental conditions and natural intronic polymorphisms. Cold exposure influences *COOLAIR* splicing (Swiezewski et al., 2009). For example, class I *COOLAIR* RNAs increase more rapidly than do class II ones during vernalisation (Csorba et al., 2014; Eom et al., 2018). *Arabidopsis* accessions show variable *COOLAIR* splicing patterns that affect FLC expression and flowering time. A single nucleotide polymorphism (SNP) specifically regulates *COOLAIR* AS (Li et al., 2015). This SNP is located next to the acceptor splice site of the intron of class IIi *COOLAIR*. In later flowering accessions, such as Var2-6 and Eden-1, this SNP reduces use of the splice acceptor site of the class IIi *COOLAIR* intron, leading to a shift to a downstream distal splice acceptor site and the inclusion of an internal exon. This change of splicing site produces isoforms with altered secondary structure and upregulates *FLC* expression (Li et al., 2015; Hawkes et al., 2016).

## Splicing Factors Regulate the Processing of *FLC* Transcript

Splice sites, including 5′ donor splice site, branch point site, polypyrimidine tract and 3′ acceptor splice site, in pre-mRNA introns are precisely recognized by some splicing factors. Numerous splicing factors, such as BRR2, SC35 and SC35-like (SCL), RZ-1B and RZ-1C, are involved in the splicing of *FLC* in *Arabidopsis* flowering transition (**Table 1**). BRR2, an ATP-dependent RNA helicase, is an integral component of the U5 snRNP that is required for the activation of the spliceosome complex (Raghunathan and Guthrie, 1998). A missense mutation in *Arabidopsis BRR2a* results in defective *FLC* splicing and reduced *FLC* transcript levels (Mahrez et al., 2016). SC35 is a serine/arginine-rich (SR) protein that functions in the selection of splice sites (Valcárcel and Green, 1996). SC35 and SC35-like (SCL) proteins in *Arabidopsis* simultaneously modulate the splicing and transcription of *FLC* (Yan et al., 2017). *Arabidopsis* RZ-1B and RZ-1C, two heterogeneous nuclear ribonucleoproteins (hnRNPs), regulate *FLC* splicing and transcription by directly interacting with the SR protein (Wu et al., 2016). Interestingly, the retention of *FLC* introns 1, 5 and 6 in the *brr2a* mutant increases and the splicing efficiency of *FLC* intron 1 decreases in *rz-1b* rz*-1c* double mutants. These findings suggest that BRR2a, RZ-1B and RZ-1C promote the splicing of *FLC* introns. By contrast, the splicing efficiency of intron 1 in *FLC* increases compared with that in wild-type seedlings in quintuple mutants of *SC35* and *SCL* genes, indicating that SC35 and SCL proteins inhibit the splicing of the first intron of *FLC*. The splicing efficiency of *FLC* introns is also inhibited by KHZ1 and KHZ2, two RNA-binding proteins containing CCCH zinc-finger and K homology (KH) domain (Yan et al., 2019).

**Table 1:** Proteins involved in pre-mRNA splicing of *FLC* and *COOLAIR*.

Proteins	Type of Proteins	Splicing	References
U2AF65a, U2AF65b	Subunits of U2 auxiliary factors	*FLC* introns	Park et al., 2019a; Xiong et al., 2019a
SC35, SCL28,30,30A,33	SR proteins	*FLC* intron 1	Yan et al., 2017
BRR2a	U5 snRNP	*FLC* introns 1, 5 and 6	Mahrez et al., 2016
RZ-1B, RZ-1C	hnRNP proteins	*FLC* intron 1	Wu et al., 2016
ABH1/CBP80, CBP20	CAP-binding complex subunits; Interaction proteins of splicing factor	*FLC* introns	Geraldo et al., 2009; Kuhn et al., 2017
KHZ1, KHZ2	RNA-binding proteins	*FLC* introns	Yan et al., 2019
PRP8	Core spliceosome component	*COOLAIR* introns	Marquardt et al., 2014
NSRa	Nuclear speckle RNA binding protein	*COOLAIR* introns	Bazin et al., 2018

U2 auxiliary factor (U2AF) regulates flowering *via* modulating *FLC* splicing. U2AF65, a large subunit of *U2AF* in mammalians, recognises and binds to the 3′ polypyrimidine tract of introns (Wang et al., 2008; Shao et al., 2014). The binding site of U2AF65 with RNA is regulated and shifted in noncanonical introns (Shao et al., 2014; Howardet al., 2018). The genes *AtU2AF65a* and *AtU2AF65b* encode the *U2AF* large subunit in *Arabidopsis* (Jang et al., 2014). AtU2AF65b plays roles in regulating flowering transition by splicing the introns 1 and 6 of *FLC* (Xiong et al., 2019a) (**Table 1**). *AtU2AF65b* expression is responsive to ABA, by which *AtU2AF65b* is involved in ABA-regulated flowering. *AtU2AF65a is* also *implicated in FLC splicing (* Park et al., 2019a *). Strikingly, the* loss-of-function mutants of *AtU2AF65a* and *AtU2AF65b* display opposite flowering phenotypes. Mutations in *atu2af65a* cause late flowering, whereas *AtU2AF65b* mutants exhibit early flowering (Park et al., 2019a; Xiong et al., 2019a). The differences in the noncanonical splicing variants between *atu2af65a* and *atu2af65b* null mutants (Park et al., 2019a) indicate that AtU2AF65a and AtU2AF65b might recognise different *FLC* introns.

In addition, some proteins play roles in RNA splicing by interaction with splicing factors. For example, the mRNA cap-binding complex (CBC) is involved in modulating pre-mRNA splicing activities *via* interaction with the splicing factors that recognise the 5′ splice site of the cap proximal intron (Izaurralde et al., 1994; Lewis et al., 1996). The CBP80/ABA Hypersensitive 1 (ABH1) and CBP20 are the large and small subunits of CBC protein complex in *Arabidopsis*, respectively (Hugouvieux et al., 2001). Knockout mutants of *ABH1*/*CBP80* and *CBP20* showing early-flowering phenotypes result from the defective splicing of *FLC* introns, especially the large first intron (Kuhn et al., 2007) (**Table 1**). In the *abh1* knockout mutant, the most prominent products are the splice intermediates containing the first intron, causing the downregulation of *FLC* transcript and the early-flowering phenotypes (Kuhn et al., 2007). Similarly, *CBP20* null mutation also results in increased unspliced–spliced ratio of *FLC* introns and low *FLC* mRNA levels (Geraldo et al., 2009).

To date, little is known about the splicing regulation of *COOLAIR* transcripts mediated by splicing factors. Only one splice factor, PRP8, a core spliceosome component, is found to function in modulating *COOLAIR* RNAs splicing (Marquardt et al., 2014) (**Table 1**). PRP8 is specifically required for the splicing of antisense transcripts *COOLAIR* but not for that of *FLC* sense transcripts. Single-base mutations of *PRP8* reduce the splicing efficiency of *COOLAIR* introns, especially class Ii introns. The decreased splicing efficiency of *COOLAIR* class Ii reduces proximal poly(A) site usage, leading to increased H3K4me2 and the transcriptional upregulation of *FLC* expression (Liu et al., 2010; Marquardt et al., 2014). NSRa, as a nuclear speckle RNA binding protein, modules flowering time through regulation of the *COOLAIR* AS (**Table 1**). Only distal variants are decrease in the *nsra* mutant. This change in relative variant usage of proximal-distal RNAs leads to a down-regulation of *FLC* mRNA and an early flowering phenotype of *nsra* mutant (Bazin et al., 2018).

## Conclusion and Perspective

FLC is a key inhibitor in flowering transition in *Arabidopsis* and other dicots (Michaels and Amasino, 1999; Sheldon et al., 1999; Helliwell et al., 2006; Yuan et al., 2009; Wu et al., 2012). Therefore, the regulation of FLC is central to the transition to flowering in these plants. The AS of *FLC* sense and antisense transcripts is a critical step for *FLC* expression regulation. In *Arabidopsis*, multiple spliced variants of *FLC* sense and antisense transcripts have been determined (Kuhn et al., 2007; Severing et al., 2012; Li et al., 2015; Hawkes et al., 2016; Park et al., 2019a); however, we know little about functions *in vivo* of these spliced isoforms, especially, the *COOLAIR*-mediated regulation of switching of chromatin states at *FLC* and processing of *FLC* sense transcripts (**Figure 1B**). The further investigation will focus on how regulation of *COOLAIR* AS is linked to *FLC* chromatin modifications in response to external and internal influences.

Alternative splicing is regulated by splicing factors; however, only few splicing factors have been identified to be involved in the AS of *FLC*, especially those in the AS of *COOLAIR* transcripts. The identification of splicing factors and their functions is important for understanding the AS regulation of *FLC* sense and antisense transcripts in flowering transition. Additionally, the patterns of *FLC* AS is altered in response to environmental and signal stimuli (Swiezewski et al., 2009; Hornyik et al., 2010; Liu et al., 2010; Xiong et al., 2019a). Thus, the mechanisms by which the activities of splicing factors are regulated in response to external and internal cues must be investigated to study the AS of *FLC* sense and antisense transcripts in flowering regulation.

In agriculture, flowering is a prerequisite for crop production. Changes in the splicing patterns of *FLC* sense and antisense transcripts have enabled adaptation in response to changing environment for *Arabidopsis* accessions. Moreover, cold-induced sense and antisense RNAs of *FLC* are evolutionarily conserved in *Arabidopsis* perennial relatives and sugar beet (Reeves et al., 2007; Castaings et al., 2014; Li et al., 2015; Hawkes et al., 2016). Therefore, the molecular dissection of the diversity in splicing patterns of *FLC* across natural populations of *Arabidopsis* provides an important insight into how splicing regulation influences the switch from vegetative to reproductive growth. These findings propose a possible application for cultivating new varieties and augmenting the control of flowering time to adapt the environmental changes *via* modulating *FLC* expression in some crops, such as *Brassicaceae*.

## Author Contributions

X-LW designed the concept, organized and drafted the text. H-DQ performed the meta-analysis, drafted text, and prepared figures. H-DQ and YL edited the manuscript with help of Q-PR, Y-YW and FX.

## Funding

This work was supported by National Natural Science Foundation of China (31970330) and Major Basic Research Projects of Shandong Natural Science Foundation (ZR2018ZC08N3).

## Conflict of Interest

The authors declare that the research was conducted in the absence of any commercial or financial relationships that could be construed as a potential conflict of interest.
